# Targeted Covalent Inhibitors in Drug Discovery, Chemical Biology and Beyond

**DOI:** 10.3390/ph17020206

**Published:** 2024-02-05

**Authors:** Ricardo A. M. Serafim, Matthias Gehringer, Chiara Borsari

**Affiliations:** 1 Department of Pharmaceutical/Medicinal Chemistry, Institute of Pharmaceutical Sciences, Eberhard Karls University Tübingen, 72076 Tübingen, Germany; 2 Cluster of Excellence iFIT (EXC 2180) ‘Image-Guided & Functionally Instructed Tumor Therapies’, University of Tübingen, 72076 Tübingen, Germany; 3 Department of Pharmaceutical Sciences, University of Milan, 20133 Milan, Italy

Covalent inhibitors have experienced a revival in medicinal chemistry and chemical biology in recent decades [1,2]. Although such compounds have a long and successful history in drug discovery, as exemplified by covalently acting drugs like aspirin or β-lactam antibiotics, the deliberate design of compounds with a covalent mode of action has long been avoided due to concerns about safety issues linked to the permanent modification of non-target proteins or immunogenicity triggered by haptenization [3,4]. In the last two decades, the manifold possibilities arising from the covalent attachment of inhibitors or other types of pharmacological modulators to their targets has led to a resurgence of interest in covalency within drug discovery and beyond. Since the early 2000s, we have seen a steep increase in publication and patenting activity in this field alongside the approval of various rationally developed covalent drugs, so-called targeted covalent inhibitors, or TCIs [5]. For example, the first two covalent protein kinase inhibitors, Afatinib [6] and Ibrutinib [7], were introduced to the market in 2013, and eight other protein kinase TCIs have followed since then [8,9,10]. Importantly, many of the initial safety concerns do not seem to have substantiated, suggesting that the field of covalent drug discovery will continue growing in the coming years [2].

TCIs engage their biological targets through the reversible or irreversible formation of a covalent bond, which occurs after the proper positioning of the inhibitor’s reactive group (typically a weak electrophile, the so-called “warhead”) in relation to a specific target amino acid [11]. Even though the cysteine-targeting α,β-unsaturated amides are the dominant electrophiles in TCIs, the warhead toolbox is expanding [12,13], and probes with different warhead chemotypes are being discovered. The covalent binding mechanism confers special properties, most notably an increased and time-dependent potency [14]. Through the clever selection of a non-conserved target amino acid in conjunction with a low-reactivity warhead and a suitable reversible binding element, covalency can also be used as a means to increase selectivity [15]. These features have been exploited in the generation of TCIs targeting “undruggable” proteins, as impressively highlighted by the development of the KRAS^G12C^ inhibitors like Sotorasib (AMG 510) [16] and Adagrasib (MRTX849) [17]. Importantly, the irreversible engagement of targets with a low re-synthesis rate may also provide an opportunity to achieve prolonged pharmacological effects at lower and/or less frequent drug doses, thus reducing exposure and the potential for toxicities. Due to these particular properties, as well as their ability to label proteins with various tags, covalent targeting strategies have also recently attracted much interested in the field of chemical biology, for example, in the framework of chemoproteomic approaches [1] or in the discovery of chemical probes [18]. On the other hand, it is also clear that TCIs require a different repertoire of methods for their characterization and must meet a different set of criteria to be amenable as high-quality chemical probes or drugs [19,20].

In this Special Issue on “Targeted Covalent Inhibitors in Drug Discovery, Chemical Biology and Beyond”, we aimed to collect manuscripts highlighting the recent advances in covalent drug discovery; the toolbox of warhead chemistries and their computational investigation; novel approaches for covalent hit and lead generation; and their application potential. The issue features three original articles and six reviews from authors with a diverse range of expertise and cultural backgrounds, as reported below.

In their research paper, Koch and colleagues designed and optimized a series of pyridinylimidazole derivatives as “turn-on” c-Jun N-terminal kinase 3 (JNK3) covalent inhibitors by applying a photocaging approach (*contribution 1*). The most promising acrylamide-based inhibitor was equipped with a photocleavable protecting group at the hinge-binding motif, leading to a less active compound, which undergoes deprotection when exposed to UV-light irradiation and consequently recovers the target engagement in living cells. Therefore, the authors successfully confirmed the application of the photocaging method to pharmacologically control JNK3 activity in a cellular environment.

Pillaiyar and colleagues reported their work on the discovery of natural products as inhibitors of the SARS-CoV-2 main protease (M^pro^). In the search for novel M^Pro^ inhibitor chemotypes, they screened a variety of electrophilic natural products to discover hits with low micromolar activities, including several compounds showing antiviral properties without cytotoxicity. While they used molecular docking to deduce binding modes for several inhibitors, a co-crystal structure of the polyphenol Robinetin and M^Pro^ unambiguously revealed its covalent mechanism, wherein the pyrogallol group attaches to the catalytic Cys145 via an oxidative mechanism (*contribution 2*).

A team at Boehringer Ingelheim reported the application of their BIreactive approach to predict the reactivity of propynamide derivatives, which are included in several clinical candidates and approved drugs. Here, Hermann and colleagues used different in silico parameters to describe the properties of propynamide: the electrophilicity index, adduct formation, and transition-state energies (*contribution 3*). They found out that the adduct formation energy and the transition-state energy can be exploited to reliably predict the *in vitro* reactivity with glutathione, which is used as a cysteine surrogate. In contrast, the electrophilicity index displayed limitations in predicting the reactivity of substituted propynamides.

The featured review article from McAulay and colleagues focuses on the application of fragment-based drug discovery (FBDD) approaches to the field of covalent ligands (*contribution 4*). The authors provided an overview of the methods exploited to assess intrinsic warhead reactivity as a pivotal requirement to characterize an electrophilic fragment library. The advantages of covalent FBDD over “binder-first” approaches are outlined, and a comprehensive expert overview on successful case studies is provided. The authors present a detailed description on the FBDD approaches behind the discovery of covalent inhibitors for previously “undruggable” or complex protein targets, including KRAS^G12C^, SARS-CoV-2, BRD4-BET2, and Pin1.

The review article by Ferreira and colleagues explores the development of covalent inhibitors in the field of neglected parasitic diseases (*contribution 5*). The authors showcase the design and SAR aspects of several TCIs containing a variety of warheads, from traditional α,β-unsaturated Michael acceptors to boronate derivatives, against protein targets of Chagas disease, human African trypanosomiasis (HAT), and Malaria. Beyond the well-known inhibitors targeting the catalytic cysteines of proteases, such as cruzain and falcipain from *Trypanosoma cruzi* and *Plasmodium falciparum*, respectively, the authors also covered, for instance, inhibitors binding threonine residues from the active site of the *P. falciparum* proteasome. Additionally, they highlight some important pharmacokinetics properties for many of the described compounds. 

Schaefer and Cheng provide an up-to-date overview of the advances in covalent drug discovery (*contribution 6*). They include a historical perspective with milestones and showcase the evolution of the field from early discoveries to its recent resurgence. They highlight various functional groups and binding mechanisms of both historic examples (like aspirin and penicillin) and more recently developed TCIs and provide a concise overview on the design, properties, and characterization of TCIs. Beyond this, the article covers recent developments, like covalent SARS-CoV-2 M^pro^ inhibitors and covalent PROTAC degraders.

The characterization of the exact nature of a covalent protein–drug interaction and the kinetics of covalent adduct formation is extremely important for the investigation of covalent ligands. In their featured article, Mons, Kim, and Mulder provide a comprehensive expert overview of technologies for the direct detection of covalent protein–drug adducts (*contribution 7*). These include, amongst others, label-free techniques like mass spectrometry, protein crystallography, or monitoring changes in spectroscopic ligand properties upon adduction, but also chemoproteomic and NMR-based approaches employing modified versions of the covalent drug. The article includes an elaborate discussion of the strengths and limitations of these techniques and their ability to investigate properties like inactivation kinetics and reversible covalency, providing an excellent insight into which techniques are best applied to a particular problem.

The concise review by Zhao and Bourne outlines the recent advancements in covalent kinase inhibitors (CKIs), which hold promise due to their exceptional selectivity and affinity. The warhead chemical space of CKIs is analyzed and matched with the corresponding targeted amino acid residue. Some examples of reversible and irreversible covalent molecules are reported to showcase the impressive progress in the field (*contribution 8*). 

The featured review article provided by Lee and Park spans from classical and recent FDA-approved covalent drugs targeting catalytic amino acids to modern TCIs designed to interact with non-catalytic residues (*contribution 9*). To highlight the applicability of TCIs to targets other than enzymes, they extended their overview to covalent inhibitors targeting protein–protein interactions, such as the drug Selinexor, which covalently binds to the nuclear export protein XPO1. In this context, examples of the covalent allosteric modulation of PPIs are also presented. Moreover, the authors give expert insights into valuable techniques which may continuously help the upcoming evolution of the field. 

Taken together, these papers fittingly represent the broad spectrum of work that is currently being performed in the field of TCIs and other covalent modulators. We hope that this Special Issue helps to inspire and educate the scientific community and triggers innovative ideas in the covalent inhibitor landscape.

## List of Contributions

1. Hoffelner, B.S.; Andreev, S.; Plank, N.; Koch, P. Photocaging of Pyridinylimidazole-Based Covalent JNK3 Inhibitors Affords Spatiotemporal Control of the Binding Affinity in Live Cells. *Pharmaceuticals* **2023**, *16*, 264. https://doi.org/10.3390/ph16020264.
2. Krüger, N.; Kronenberger, T.; Xie, H.; Rocha, C.; Pöhlmann, S.; Su, H.; Xu, Y.; Laufer, S.A.; Pillaiyar, T. Discovery of Polyphenolic Natural Products as SARS-CoV-2 M(pro) Inhibitors for COVID-19. *Pharmaceuticals* **2023**, *16*, 190. https://doi.org/10.3390/ph16020190.
3. Hermann, M.R.; Tautermann, C.S.; Sieger, P.; Grundl, M.A.; Weber, A. BIreactive: Expanding the Scope of Reactivity Predictions to Propynamides. *Pharmaceuticals* **2023**, *16*, 116. https://doi.org/10.3390/ph16010116.
4. McAulay, K.; Bilsland, A.; Bon, M. Reactivity of Covalent Fragments and Their Role in Fragment Based Drug Discovery. *Pharmaceuticals* **2022**, *15*, 1366. https://doi.org/10.3390/ph15111366.
5. Alves, E.T.M.; Pernichelle, F.G.; Nascimento, L.A.; Ferreira, G.M.; Ferreira, E.I. Covalent Inhibitors for Neglected Diseases: An Exploration of Novel Therapeutic Options. *Pharmaceuticals* **2023**, *16*, 1028. https://doi.org/10.3390/ph16071028.
6. Schaefer, D.; Cheng, X. Recent Advances in Covalent Drug Discovery. *Pharmaceuticals* **2023**, *16*, 663. https://doi.org/10.3390/ph16050663.
7. Mons, E.; Kim, R.Q.; Mulder, M.P.C. Technologies for Direct Detection of Covalent Protein-Drug Adducts. *Pharmaceuticals* **2023**, *16*, 547. https://doi.org/10.3390/ph16040547.
8. Zhao, Z.; Bourne, P.E. Systematic Exploration of Privileged Warheads for Covalent Kinase Drug Discovery. *Pharmaceuticals* **2022**, *15*, 1322. https://doi.org/10.3390/ph15111322.
9. Lee, J.; Park, S.B. Extended Applications of Small-Molecule Covalent Inhibitors toward Novel Therapeutic Targets. *Pharmaceuticals* **2022**, *15*, 1478. https://doi.org/10.3390/ph15121478.
